# Evaluation of Non-Invasive Biological Samples to Monitor *Staphylococcus aureus* Colonization in Great Apes and Lemurs

**DOI:** 10.1371/journal.pone.0078046

**Published:** 2013-10-21

**Authors:** Frieder Schaumburg, Lawrence Mugisha, Peter Kappeller, Claudia Fichtel, Robin Köck, Sophie Köndgen, Karsten Becker, Christophe Boesch, Georg Peters, Fabian Leendertz

**Affiliations:** 1 Institute of Medical Microbiology, University Hospital Münster, Münster, Germany; 2 College of Veterinary Medicine, Animal Resources and Biosecurity, Makerere University, Kampala, Uganda; 3 Conservation & Ecosystem Health Alliance, Kampala, Uganda; 4 Behavioral Ecology and Sociobiology Unit, German Primate Center, Göttingen, Germany; 5 Institute of Hygiene, University Hospital Münster, Münster, Germany; 6 Project Group Epidemiology of Highly Pathogenic Microorganisms, Robert Koch-Institut, Berlin, Germany; 7 Department of Primatology, Max-Planck-Institute for Evolutionary Anthropology, Leipzig, Germany; Universitätsklinikum Hamburg-Eppendorf, Germany

## Abstract

**Introduction:**

Reintroduction of endangered animals as part of conservational programs bears the risk of importing human pathogens from the sanctuary to the natural habitat. One bacterial pathogen that serves as a model organism to analyze this transmission is *Staphylococcus aureus* as it can colonize and infect both humans and animals. The aim of this study was to evaluate the utility of various biological samples to monitor *S. aureus* colonization in great apes and lemurs.

**Methods:**

Mucosal swabs from wild lemurs (n=25, Kirindy, Madagascar), feces, oral and genital swabs from captive chimpanzees (n=58, Ngamba and Entebbe, Uganda) and fruit wadges and feces from wild chimpanzees (n=21, Taï National Parc, Côte d’Ivoire) were screened for *S. aureus*. Antimicrobial resistance and selected virulence factors were tested for each isolate. Sequence based genotyping (*spa* typing, multilocus sequence typing) was applied to assess the population structure of *S. aureus*.

**Results:**

Oro-pharyngeal carriage of *S. aureus* was high in lemurs (72%, n=18) and captive chimpanzees (69.2%, n=27 and 100%, n=6, respectively). Wild chimpanzees shed *S. aureus* through feces (43.8, n=7) and fruit wadges (54.5, n=12). Analysis of multiple sampling revealed that two samples are sufficient to detect those animals which shed *S. aureus* through feces or fruit wadges. Genotyping showed that captive animals are more frequently colonized with human-associated *S. aureus* lineages.

**Conclusion:**

Oro-pharyngeal swabs are useful to screen for *S. aureus* colonization in apes and lemurs before reintroduction. Duplicates of stool and fruit wadges reliably detect *S. aureus* shedding in wild chimpanzees. We propose to apply these sampling strategies in future reintroduction programs to screen for *S. aureus* colonization. They may also be useful to monitor *S. aureus* in wild populations.

## Introduction

Wildlife sanctuaries contribute to the conservation of endangered animals by providing treatment, rehabilitation and long-term care [1]. Recently, it has been demonstrated, that drug-resistant human *Staphylococcus aureus* can be transmitted from care workers and veterinarians to chimpanzees (*Pan troglodytes*) and gorillas (*Gorilla gorilla*), as shown in two sanctuaries in Uganda and Zambia [2] or in a research center in Gabon [3]. Apart from colonization, it was reported that transmitted *S. aureus* has led to severe and even lethal infection of one gorilla in the Gabonese center [3] highlighting the threat of this “humanosis” in apes. It has been reported that *S. aureus* can cause sepsis, skin and soft tissue infection, visceral abscess, air sacculitis and meningitis in monkeys and apes [3–5].

 It is likely, that fatal *S. aureus* infections could not only occur in captive animals but also in wild conspecifics. Most sanctuaries consider reintroduction as an important tool for the conservation of endangered animals [1]. This bears the risk to introduce human adapted pathogens into the wild population where surveillance and infection control measures are hardly feasible [2,6]. 

A surveillance strategy for pathogens can therefore be used to assess the risk caused by re-introduction of captive wild animals into their natural habitat. Ideally, specimens used for surveillance should be non-invasive to avoid narcotization of wild animals for sampling of mucosal swabs. While the anterior nares are known as principle habitat of *S. aureus*, the main colonization sites for monkeys and apes are largely unknown.

Recently it has been shown that a number of viruses and bacteria can be monitored in fecal and urine samples, e.g. respiratory viruses, SIV and plasmodium [7,8] or antibodies to simian T-lymphotropic virus [9]. Similarly, fecal samples were used to show that *Escherichia coli* can be transmitted between humans, gorillas and livestock if their habitats overlap [10]. Recently, we also showed that chewed spit-out remains (fruit wadges) from chimpanzees are suitable to analyze oro-pharyngeal carriage of *S. aureus* [11].

The objectives of our study were (i) to identify the most suitable anatomical sites or specimens to assess *S. aureus* colonization among lemurs and apes as exemplified by samples from captive chimpanzees (Uganda), wild chimpanzees (Côte d’Ivoire) and wild lemurs (red fronted lemurs and Verreaux’s sifakas, Madagascar) and (ii) to assess the detection rate of *S. aureus* from non-invasive samples (stool and fruit wadges) from chimpanzees. 

## Methods

### Ethics

The samples from sanctuary chimpanzees were collected as part of routine health check-ups and from lemurs in the course of radio collaring captures in accordance with international guidelines and under general anesthesia. No animal was sacrificed. Permission to take samples from chimpanzees was issued by the Chimpanzee Sanctuary and Wildlife Conservation Trust, the Uganda Wildlife Authority, The Uganda National Council of Science and Technology and CITES authorities of Uganda and the “L’ Office Ivoirien des Parcs et Réserves”. From wild chimpanzees only non-invasive samples were taken without disturbing their natural behavior [12].

All procedures on lemurs were part of routine surveillance and have been performed in accordance with the European animal welfare regulations (EU directive 2010/63 EU, German Animal Welfare Act) and permission of the authorities in Madagascar (“Direction Générale des Forets, Direction de la Conservation de la Biodiversité et du Système des Aires Protégées”: No: 069/13/MEF/Sg/DGF/DCB.SAP/SCBE). 

In addition, the animal welfare officer of the German Primate Center, Göttingen, Germany, agreed prior to the capture procedure of the lemurs, no ethical approval was required. The German Primate Center is registered and authorised by the local and regional veterinary governmental authorities. Persons, who are carrying out procedures on animals, taking care for the animals or design the projects, have the authorisation by the veterinary authorities. Bacterial samples were taken under appropriate narcosis (ketamine) under supervision of a veterinarian. 

### Sample collection

Samples from wild lemurs (red-fronted lemurs, *Eulemur rufifrons* and Verreaux’s sifaka, *Propithecus verreauxi*) living in Kirindy Forest Reserve, Madagascar (n=25) were obtained from nares, rectum, throat and vagina while animals were caught for radio collaring purpose and health assessment in 2012. All samples were stored on cotton swabs in Amies transport medium at room temperature until culture within one week. Stool samples, oral and genital swabs were obtained from chimpanzees (*Pan troglodytes schweinfurthii*) from Ngamba Island Sanctuary, Ngamba, Uganda (n=46) and Uganda Wildlife Centre, Entebbe, Uganda (n=12), collected during routine yearly health checks in 2007 and 2011. These samples were stored in STTG-medium. All chimpanzees from Uganda were considered to be captive but wild borne.

Samples from wild chimpanzees (*P. t. verus*) living in Taï National Park, Côte d’Ivoire were derived from a retrospective sample collection in 2008 [11]. Samples (n=21) consisted of fruit wadges, preserved in STTG-medium as well as feces collected within three minutes after defecation in the course of routine health studies [12]. All samples were stored at minimum -20°C until microbiological culture.

### Identification and antimicrobial susceptibility testing

Samples were streaked on Columbia blood agar plates supplemented with an aztreonam disk, SAID and colistin-aztreonam agar plates (both bioMérieux, Marcy l’Etoile, France). Catalase test and a latex agglutination test (Pastorex Staph-Plus, BioRad, Marnes la Coquette, France) were applied for presumptive *S. aureus* colonies. Species identification of *S. aureus* and antimicrobial susceptibility test was performed using VITEK 2 automated system (bioMérieux). Species was confirmed by the gene detection of the thermostable nuclease (nuc) [13].

### Virulence factors

Genes encoding the virulence factors Panton-Valentine leukocidin (*lukS*-PV/*lukF*-PV), staphylococcal pyrogenic toxin superantigen (PTSAg) genes and exfoliative toxins were detected by PCR as published [14,15] . 

### Phylogenetic analysis

All isolates were subjected to *S. aureus* protein A typing (*spa* typing) [16]. Multilocus sequence typing (MLST) was done exemplarily for one isolate of each *spa* type in each animal group [17]. To correct for sampling bias, only one isolate per *spa* type was included from each carrier.

## Results

### Carriage pattern

In total, 100 *S. aureus* isolates were found; all harbored the *nuc* gene. To calculate colonization rates in the different animal groups, we included only one sample (first sample) per anatomical site in case of multiple samples from one animal. The oro-pharyngeal *S. aureus* colonization rate was high in captive chimpanzees (69.2%, n=27 and 100%, n=6) from Uganda and wild lemurs (72%, n=18, table 1). In contrast, rectal colonization was low in lemurs (8.7%, n=2) and not detected in captive chimpanzees. While vaginal colonization in female lemurs was low (11.1%, n=2), captive female chimpanzees were vaginal carriers of *S. aureus* in 50% (n=2, Entebbe) or 80% (n=16, Ngamba table 1). 

**Table 1:** Colonization pattern of *S. aureus* in lemurs and chimpanzees.

Characteristics		Species studied			
		Lemurs	Chimpanzees		
Country		Madagascar	Uganda		Côte d’Ivoire
Study site		Kirindy Forest	Ngamba	Entebbe	Taï National Parc
Life		Wild	Captive	Captive	Wild
No. of animals		25	46	12	21
*S. aureus* colonization^1^	Nasal	36.8 (7)	-	-	-
	Oro-pharyngeal	72.0 (18)	69.2 (27)	100 (6)	-
	Rectal	8.7 (2)	-	0 (0)	-
	Vaginal	11.1 (2)	80.0 (16)	50 (2)	-
	Fecal	-	25 (2)	0 (0)	43.8 (7)
	Fruit wadges	-	-	-	54.5 (12)

^1^ Figures are % (n) of positive samples from the respective anatomical site/sample material

### Detection rate of multiple sampling

We assessed the number of consecutive stool samples which are necessary to reliably detect the colonization status of the animal.

Stool samples were collected from chimpanzees (n=29) in Uganda and Côte d’Ivoire at baseline (n=29) after an interval of 15 days (median, range: 2-123, n=15) and 23 days (median, range: 3-127, n=13). Table 2 shows the proportion of positive stool cultures and the cumulative of positive stool cultures in chimpanzees. We detected 84.6% of *S. aureus* carriers (n=11) when analyzing one stool sample from each individual. All carriers would be detected when analyzing two samples. Similarly, we analyzed fruit wadges from 21 chimpanzees (Côte d’Ivoire) which were taken at baseline (n=21), after 1 day (median, range: 0.5-3, n=5) and after 11 days (median, range: 9-13, n=4). Microbiological culture of only one sample would detect 85.7%, a second sample would detect accumulatively 100% of those chimpanzees which shed *S. aureus* through fruit wadges (table 2). Similarly to stool samples, a third sample of fruit wadges would not increase the detection rate of *S. aureus* (table 2).

**Table 2:** The cumulative of positive *S. aureus* cultures from stool samples and fruit wadges at different time points in chimpanzees.

Time point	Stool		Fruit wadges	
	Positive culture (%)	Cumulative positive cultures (%)	Positive culture (%)	Cumulative positive cultures (%)
1	11/29 (37.9)	11 (84.6)	12/21 (57.1)	12 (85.7)
2	4/15 (26.7)	13 (100)	3/5 (60.0)	14 (100)
3	2/13 (15.4)	13 (100)	3/4 (75.0)	14 (100)

### Population structure

We performed sequence based genotyping of all isolates to investigate the genetic background of *S. aureus* from the four study sites. The majority of MLST STs were solely found in one studied site. Only two STs were found in two different regions: ST1 (t127, t10694, Kirindy, n=6 and Taï National Parc, n=3) and ST188 (t189, Kirindy, n=10 and Ngamba, n=3). A predominance of certain *S. aureus* lineages were found in Kirindy (ST188, t189, n=10), Ngamba (ST2168, t1247, n=18 and ST80, t934, n=18) and Entebbe (ST6, t2360, n=6). While colonization rates were similar in sifakas (81%, n=13) and red-fronted lemurs (67%, n=6), we found a species related distribution of genotypes among lemurs: sifakas were colonized with *S. aureus* belonging to *spa* types t127 (ST1, n=1), t189 (ST188, n=10), t493 (ST182, n=2), t1429 (ST2436, n=1, Table 3). Red-fronted lemurs were colonized with isolates belonging to *spa* type t10694 (ST1, n=5) and t10695 (ST2435, n=5). One sifaka carried an isolate that was only found in red-fronted lemurs belonging to *spa* type t10694 (ST1).

**Table 3:** Population structure, antibiotic resistance and selected virulence factors of *S. aureus* from lemurs and chimpanzees.

Site/Country	Species	Genotypes		Antimicrobial resistance, n (%)			Virulence factors, n (%)			Number
		ST	*spa* type (n)	Penicillin	Tetracycline	SXT^1^	PVL	SEA	ETD	
Kirindy forest /Madagascar	Lemurs	ST1	**t10694** (5), t127 (1)	1 (16.7)	0 (0)	0 (0)	0 (0)	0 (0)	0 (0)	6
		ST182	t493 (2)	1 (50)	0 (0)	0 (0)	0 (0)	0 (0)	0 (0)	2
		ST188	t189 (10)	0 (0)	0 (0)	0 (0)	0 (0)	0 (0)	0 (0)	10
		ST2435	**t10695** (2)	0 (0)	0 (0)	0 (0)	0 (0)	0 (0)	0 (0)	2
		ST2436	t1429 (1)	0 (0)	0 (0)	0 (0)	0 (0)	0 (0)	0 (0)	1
Ngamba Island/Uganda	Chimpanzees	ST80	t934 (18)	0 (0)	15 (83.3)	0 (0)	18 (100)	0 (0)	18 (100)	18
		ST188	t189 (3)	0 (0)	0 (0)	0 (0)	0 (0)	0 (0)	0 (0)	3
		ST2126	t084 (1)	1 (100)	0 (0)	0 (0)	0 (0)	0 (0)	0 (0)	1
		ST2168	t1247 (18)	18 (100)	0 (0)	0 (0)	0 (0)	0 (0)	0 (0)	18
		ST2178	t2864 (1)	1 (100)	0 (0)	1 (100)	1 (100)	0 (0)	0 (0)	1
Taï National Parc/Côte d’Ivoire	Chimpanzees	ST1	t127 (1), t1931 (1), **t6963** (1)	0 (0)	0 (0)	0 (0)	0 (0)	0 (0)	0 (0)	3
		ST45	t015 (1)	0 (0)	0 (0)	0 (0)	0 (0)	0 (0)	0 (0)	1
		ST601	**t11388** (1), **t6960** (1)	1 (50)	0 (0)	0 (0)	0 (0)	0 (0)	0 (0)	2
		ST1928	**t6964** (4)	0 (0)	0 (0)	0 (0)	0 (0)	0 (0)	0 (0)	4
		ST2603	**t11390** (2)	2 (100)	0 (0)	0 (0)	0 (0)	0 (0)	0 (0)	2
		ST2621	**t11389** (2)	0 (0)	0 (0)	0 (0)	0 (0)	0 (0)	0 (0)	2
Uganda Wildlife Entebbe/Uganda	Chimpanzees	ST6	t2360 (6)	6 (100)	0 (0)	0(0)	0 (0)	6 (100)	2 (33.3)	6
		ST152	t355 (1)	1 (100)	0 (0)	0 (0)	1 (100)	0 (0)	0 (0)	1
		ST1292	**t11391** (1)	0 (0)	0 (0)	0 (0)	0 (0)	0 (0)	0 (0)	1
Total	-	-	-	32 (38.1)	15 (17.9)	1 (1.2)	20 (23.8)	6 (7.1)	20 (23.8)	84

Note: New, so far unknown *spa* types are in **bold**

^1^ SXT:Cotrimoxazole

### Antimicrobial resistance

The penicillin resistance in isolates from wild chimpanzees (Taï National Parc, n=14) and lemurs (Kirindy, n=21) was 21.4% (n=3) and 9.5% (n=2), respectively. In contrast, captive populations from Ngamba and Entebbe (n=49) carried more frequently isolates resistant to penicillin (55.1%, n=27), tetracycline (30.6%, n=15), erythromycin (1%, n=1), clindamycin (1%, n=1) and cotrimoxazole (2.0%, n=1). No resistance was detected against oxacillin, aminoglycosides, quinolones, linezolid, glycopeptides, fosfomycin and rifampicin. Noteworthy, virulence factors which can be associated with diseases in humans such as Panton-Valentine Leukocidine (PVL, necrotizing infections), staphylococcal enterotoxin A (gastroenteritis) or exfoliative toxin D (exfoliative dermatitis) were only found in isolates from captive chimpanzees (Entebbe, table 3).

## Discussion

Recent reports on *S. aureus* infection and colonization in chimpanzees and gorillas fueled the concern of the transmission of human pathogens such as *S. aureus* or *E. coli* to great apes [2,3,10]. Screening for potential pathogens prior to reintroduction and the surveillance of these pathogens in wild conspecifics is therefore suggested to combat “humanoses” in endangered animals [6].

In the present study, we analyzed the colonization pattern and genetic structure of *S. aureus* in lemurs, wild and captive chimpanzees in Africa. Main findings are high oro-pharyngeal colonization in lemurs and captive chimpanzees and frequent vaginal colonization in captive female chimpanzees. Duplicate samples of stool and fruit wadges from wild chimpanzees reliably detect those animals which shed *S. aureus*. These data are essential tools to monitor the anthropozoonotic acquisition of *S. aureus* both in the sanctuary setting and in the wild. 

Nasal swabs from wild lemurs revealed a nasal carriage rate of 36.7% which is similar to nasal colonization in captive macaques (*Macaca mulatta*, 39%), although they belong to a different suborder [18]. The high proportion of vaginal colonization in chimpanzees (50.0-80.0%) has been reported in captive female lion tamarins (*Leontopithecus* sp.) as well (24%) but is in contrast to 5% vaginal carriage in humans [19,20]. Similarly, oro-pharyngeal colonization was more frequent in lemurs (72%) and captive chimpanzees (69.2-100%, table 1) compared to humans (10-20%) [19]. In addition, detection rate of *S. aureus* in stools from wild chimpanzees was slightly higher than intestinal colonization rates in humans (43.8% vs. 20%) [21]. As oro-pharyngeal swabs and stool samples were not simultaneously taken, we cannot assess the correlation between oro-pharyngeal and intestinal carriage of *S. aureus* in chimpanzees. However, positive correlation between intestinal and nasal colonization is reported from humans [21]. In general, the feasibility to take mucosal swabs is constricted as aesthesia is usually necessary to be able to take nasal or oro-pharyngeal swabs from monkeys and apes even in the sanctuary setting. 

The detection of *S. aureus* from multiple stool samples and fruit wadges enabled us to calculate the needed number of consecutive samples to reliably detect *S. aureus* shedding. We showed that two samples (stool or fruit wadge) of each individual are sufficient to identify shedding of *S. aureus*. An additional third sample did not increase the detection rate. For hospitalized human patients it is known that the detection of nasal *S. aureus* colonization can be increased from 94% to 100% if a third nasal swab is taken after 24 hours [22]. 

The predominance of certain *S. aureus* lineages in Kirindy (ST188), Ngamba (ST2168 and ST80) and Entebbe (ST6) indicates a high transmissibility of these clones. While ST2168 has only been reported from captive chimpanzees so far, ST6, ST80 and ST188 are mainly prevalent in humans in Gabon and Mali [2,23,24]. It is therefore likely that human associated lineages have been transmitted to animal groups. This human to animal transmission has been shown in captive chimpanzees in Zambia and Uganda [2] and most likely occurred in a primate research center in Gabon [3]. However, the *S. aureus* lineage that caused a fatal infection in a captive gorilla in Gabon was not detected in our collection (ST72, t148) [3]. In general a trend may be visible here in the frequency of detection of likely human strains of *S. aureus* along a gradient from “no” to “intense” history of human contact (captive primates > primates captured once or every other year to exchange the radio collar> wild and never touched). Although our study provides valuable information to screen for and monitor human *S. aureus* lineages in sanctuary animals and wild conspecifics, some limitations need to be addressed. First, the small sample size and missing data on other species limit general conclusion. Second, detection rate of *S. aureus* in multiple samples from stools and fruit wadges was hampered by the high number of missing follow-up samples. It is a challenge to permanently observe wild living chimpanzees in order to collect stool samples from the respective animal in a given period of time.

In conclusion, nasal, oro-pharyngeal and vaginal swabs are useful to screen for *S. aureus* colonization in animals before reintroduction. Duplicates of non-invasive samples (stool and fruit wadges) reliably detect *S. aureus* shedding in wild chimpanzees. These data will help to establish screening and surveillance strategies for human-associated bacteria (e. g. *E. coli*) in African wildlife.

## References

[1] Faust LJ, Cress D, Farmer KH, Ross SR, Beck BB (2011) Predicting capacity demand on sanctuaries for African chimpanzees (*Pan* *troglodytes*). Int J Primatol 32: 849-864. doi:10.1007/s10764-011-9505-z.

[2] Schaumburg F, Mugisha L, Peck B, Becker K, Gillespie TR et al. (2012) Drug-resistant human *Staphylococcus* *aureus* in sanctuary apes pose a threat to endangered wild ape populations. Am J Primatol 74: 1071-1075. doi:10.1002/ajp.22067. PubMed: 22907634. 22907634

[3] Nagel M, Dischinger J, Türck M, Verrier D, Oedenkoven M et al. (2012) Human-associated *Staphylococcus* *aureus* strains within great ape populations in Central Africa (Gabon). Clin Microbiol Infect PubMed: 23398468. 10.1111/1469-0691.12119 23398468

[4] Blakeslee JR, Mcclure HM, Anderson DC, Bauer RM, Huff LY et al. (1987) Chronic fatal disease in gorillas seropositive for Simian T-lymphotropic virus I antibodies. Cancer Lett 37: 1-6. doi:10.1016/0304-3835(87)90139-X. PubMed: 2822228. 2822228

[5] Kumar S, Fox B, Owston M, Hubbard GB, Dick EJ (2012) Pathology of spontaneous air sacculitis in 37 baboons and seven chimpanzees and a brief review of the literature. J Med Primatol 41: 266-277. doi:10.1111/j.1600-0684.2012.00547.x. PubMed: 22765381. 22765381 PMC3402580

[6] Unwin S, Robinson I, Schmidt V, Colin C, Ford L et al. (2012) Does Confirmed Pathogen Transfer between Sanctuary Workers and Great Apes Mean that Reintroduction Should not Occur? Am J Primatol 74: 1076-1083. doi:10.1002/ajp.22069. PubMed: 22899168. 22899168

[7] Köndgen S, Schenk S, Pauli G, Boesch C, Leendertz F (2010) Noninvasive Monitoring of Respiratory Viruses in Wild Chimpanzees. Ecohealth, 7: 1-10. PubMed: 20665067. 20865440 10.1007/s10393-010-0340-z

[8] Gillespie TR, Nunn CL, Leendertz FH (2008) Integrative approaches to the study of primate infectious disease: Implications for biodiversity conservation and global health. Am J Phys Anthropol 137: 53-69. doi:10.1002/ajpa.20949. 19003885

[9] Leendertz FH, Boesch C, Ellerbrok H, Rietschel W, Couacy-Hymann E et al. (2004) Non-invasive testing reveals a high prevalence of simian T-lymphotropic virus type 1 antibodies in wild adult chimpanzees of the Taï National Park, Côte d'Ivoire. J Gen Virol 85: 3305-3312. doi:10.1099/vir.0.80052-0. PubMed: 15483244. 15483244

[10] Rwego IB, Isabirye-Basuta G, Gillespie TR, Goldberg TL (2008) Gastrointestinal Bacterial Transmission among Humans, Mountain Gorillas, and Livestock in Bwindi Impenetrable National Park, Uganda. Conserv Biol 22: 1600-1607. doi:10.1111/j.1523-1739.2008.01018.x. PubMed: 18717695. 18717695

[11] Schaumburg F, Alabi AS, Köck R, Mellmann A, Kremsner PG et al. (2012) Highly divergent *Staphylococcus* *aureus* isolates from African non-human primates. Environ Microbiol Rep 1: 141-146. PubMed: 23757241. 10.1111/j.1758-2229.2011.00316.x 23757241

[12] Leendertz FH, Pauli G, Maetz-Rensing K, Boardman W, Nunn C et al. (2006) Pathogens as drivers of population declines: The importance of systematic monitoring in great apes and other threatened mammals. Biol Conserv 131: 325-337. doi:10.1016/j.biocon.2006.05.002.

[13] Brakstad OG, Aasbakk K, Maeland JA (1992) Detection of *Staphylococcus* *aureus* by polymerase chain reaction amplification of the *nuc* gene. J Clin Microbiol 30: 1654-1660. PubMed: 1629319. 1629319 10.1128/jcm.30.7.1654-1660.1992 PMC265359

[14] von Eiff C, Friedrich AW, Peters G, Becker K (2004) Prevalence of genes encoding for members of the staphylococcal leukotoxin family among clinical isolates of *Staphylococcus* *aureus* . Diagn Microbiol Infect Dis 49: 157-162. doi:10.1016/j.diagmicrobio.2004.03.009. PubMed: 15246504. 15246504

[15] Becker K, Roth R, Peters G (1998) Rapid and specific detection of toxigenic *Staphylococcus* *aureus*: use of two multiplex PCR enzyme immunoassays for amplification and hybridization of staphylococcal enterotoxin genes, exfoliative toxin genes, and toxic shock syndrome toxin 1 gene. J Clin Microbiol 36: 2548-2553. PubMed: 9705390. 9705390 10.1128/jcm.36.9.2548-2553.1998 PMC105160

[16] Mellmann A, Friedrich AW, Rosenkötter N, Rothgänger J, Karch H et al. (2006) Automated DNA sequence-based early warning system for the detection of methicillin-resistant *Staphylococcus* *aureus* outbreaks. PLOS Med 3: e33. doi:10.1371/journal.pmed.0030033. PubMed: 16396609. 16396609 PMC1325475

[17] Enright MC, Day NP, Davies CE, Peacock SJ, Spratt BG (2000) Multilocus sequence typing for characterization of methicillin-resistant and methicillin-susceptible clones of *Staphylococcus* *aureus* . J Clin Microbiol 38: 1008-1015. PubMed: 10698988. 10698988 10.1128/jcm.38.3.1008-1015.2000 PMC86325

[18] van den Berg S, van Wamel WJ, Snijders SV, Ouwerling B, de Vogel CP et al. (2011) Rhesus macaques (*Macaca* *mulatta*) are natural hosts of specific Staphylococcus aureus lineages. PLOS ONE 6: e26170. doi:10.1371/journal.pone.0026170. PubMed: 22028827. 22028827 PMC3197613

[19] Wertheim HF, Melles DC, Vos MC, van Leeuwen W, van Belkum A et al. (2005) The role of nasal carriage in *Staphylococcus* *aureus* infections. Lancet Infect Dis 5: 751-762. doi:10.1016/S1473-3099(05)70295-4. PubMed: 16310147. 16310147

[20] Lilenbaum W, Moraes IA, Cardoso VS, Varges RG, Ferreira AM et al. (2006) Antibiotic resistance in Staphylococci isolated from the vaginas of captive female *Leontopithecus* (Callitrichidae Primates). Am J Primatol 68: 825-831. doi:10.1002/ajp.20282. PubMed: 16847975. 16847975

[21] Acton DS, Plat-Sinnige MJ, van Wamel W, de Groot N, an Belkum A (2009) Intestinal carriage of *Staphylococcus* *aureus*: how does its frequency compare with that of nasal carriage and what is its clinical impact? Eur J Clin Microbiol Infect Dis 28: 115-127. doi:10.1007/s10096-008-0602-7. PubMed: 18688664. 18688664

[22] Eckmanns T, Geffers C, Weist K, Rüden H (2004) One hour versus 24 h sampling intervals for the screening of patients with methicillin-resistant Staphylococcus aureus (MRSA). J Hosp Infect 57: 93-94. doi:10.1016/j.jhin.2004.01.021. PubMed: 15142723. 15142723

[23] Schaumburg F, Ngoa UA, Kösters K, Köck R, Adegnika AA et al. (2011) Virulence factors and genotypes of *Staphylococcus* *aureus* from infection and carriage in Gabon. Clin Microbiol Infect 17: 1507-1513. doi:10.1111/j.1469-0691.2011.03534.x. PubMed: 21595798. 21595798

[24] Ruimy R, Maiga A, Armand-Lefevre L, Maiga I, Diallo A et al. (2008) The carriage population of *Staphylococcus* *aureus* from Mali is composed of a combination of pandemic clones and the divergent Panton-Valentine leukocidin-positive genotype ST152. J Bacteriol 190: 3962-3968. doi:10.1128/JB.01947-07. PubMed: 18375551. 18375551 PMC2395057

