# Correction: Evaluation of Non-Invasive Biological Samples to Monitor *Staphylococcus aureus* Colonization in Great Apes and Lemurs

**DOI:** 10.1371/annotation/c2148f4d-866d-479a-b0e6-97aa6ab931f6

**Published:** 2013-11-08

**Authors:** Frieder Schaumburg, Lawrence Mugisha, Peter Kappeler, Claudia Fichtel, Robin Köck, Sophie Köndgen, Karsten Becker, Christophe Boesch, Georg Peters, Fabian Leendertz

The name of the third author was spelled incorrectly. The correct name is: Peter Kappeler. The correct citation is: Schaumburg F, Mugisha L, Kappeler P, Fichtel C, Köck R, et al. (2013) Evaluation of Non-Invasive Biological Samples to Monitor *Staphylococcus aureus* Colonization in Great Apes and Lemurs. PLoS ONE 8(10): e78046. doi:10.1371/journal.pone.0078046. 

